# A Review of Potential Therapeutic Strategies for COVID-19

**DOI:** 10.3390/v14112346

**Published:** 2022-10-25

**Authors:** Jiajia Meng, Ruijiao Li, Zhiqi Zhang, Jie Wang, Qingwen Huang, Dongxia Nie, Kai Fan, Wenbo Guo, Zhihui Zhao, Zheng Han

**Affiliations:** 1 Institute for Agro-Food Standards and Testing Technology, Shanghai Academy of Agricultural Sciences, Shanghai 201403, China; 2 School of Health Science and Engineering, University of Shanghai for Science and Technology, Shanghai 200093, China

**Keywords:** SARS-CoV-2, coronavirus, COVID-19, therapeutic strategies, pathogenesis

## Abstract

Coronavirus disease 2019 is a rather heterogeneous disease caused by severe acute respiratory syndrome coronavirus 2 (SARS-CoV-2). The ongoing pandemic is a global threat with increasing death tolls worldwide. SARS-CoV-2 belongs to lineage B β-CoV, a subgroup of Sarbecovirus. These enveloped, large, positive-sense single-stranded RNA viruses are easily spread among individuals, mainly via the respiratory system and droplets. Although the disease has been gradually controlled in many countries, once social restrictions are relaxed the virus may rebound, leading to a more severe and uncontrollable situation again, as occurred in Shanghai, China, in 2022. The current global health threat calls for the urgent development of effective therapeutic options for the treatment and prevention of SARS-CoV-2 infection. This systematic overview of possible SARS-CoV-2 therapeutic strategies from 2019 to 2022 indicates three potential targets: virus entry, virus replication, and the immune system. The information provided in this review will aid the development of more potent and specific antiviral compounds.

## 1. Introduction

Coronavirus disease 2019 (COVID-19) is caused by severe acute respiratory syndrome coronavirus 2 (SARS-CoV-2). It has spread worldwide and become a major health crisis [1,2]. Patients infected with SARS-CoV-2 display major symptoms that include fever, dyspnea, cough, and sore throat, while some patients show minor symptoms, such as dysgeusia, anosmia, gastrointestinal symptoms, headache, and skin lesions [3,4,5,6]. The condition of some patients may subsequently deteriorate rapidly, with multiple organ failure or even death within a short time [7]. Data regarding predictors of mortality in patients with COVID-19 are still scarce but are being actively investigated [8]. Person-to-person contact and respiratory droplets are the two major routes of transmission of COVID-19 infection to humans [9]. Angiotensin-converting enzyme 2 (ACE2) presented on the surface of alveolar cells in the lungs is the receptor for SARS-CoV-2 cell entry. ACE2 is also found on the mucosal cells of intestines, tubular epithelial cells of kidneys, and epithelial cells of renal tubules, which is presented as a variety of susceptible targets to SARS-CoV-2 infection [10,11]. Several studies suggested that patients with COVID-19 who use angiotensin-converting enzyme inhibitors/angiotensin receptor blockers had an increased risk of respiratory failure and death, but further randomized trials are needed to answer the question [12,13]. To date, COVID-19 has been reported from more than 200 countries; this human health threat has become the focus of global attention [14]. Actually, more than six hundred million cases have been reported with more than 6 million deaths [15].

The widespread occurrence of SARS-CoV-2 has been gradually controlled in many countries. However, once social restrictions are relaxed, the virus may rebound. This can lead to a more severe and uncontrollable situation again, such as has occurred in Shanghai, China, in 2022 [16,17]. Understanding the pathogenesis and potential targets of the virus to prevent SARS-CoV-2 infections is a global research priority. Numerous medicines have been investigated for the treatment of COVID-19 in previous studies in different parts of the world. Molnupiravir, nirmatrelvir/ritonavir, and remdesivir have been recognized as promising antiviral agents to manage COVID-19 and have been approved in different parts of the world. Moreover, some monoclonal antibodies, such as sotrovimab, casirivimab/imdevimab (Ronapreve^®^), and tixagevimab/cilgavimab (Evusheld^®^), have shown effective inhibition against COVID-19 and have permitted emergency use authorization and approval in many countries [18,19,20]. However, the benefit of these drugs is still a matter of debate due to less safety and efficacy and the variants of SARS-CoV-2.

This paper comprehensively reviews the effective recent therapeutic strategies proposed for the treatment and prevention of SARS-CoV-2 infections. Our aim is to provide insights into the development of potent and specific antivirals.

## 2. Infection Mechanisms of SARS-CoV-2

**Figure 1:**
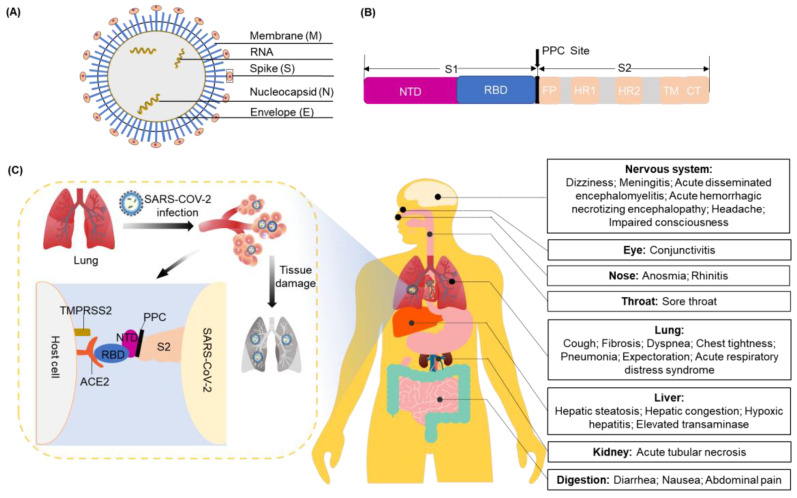
The infection of SARS-CoV-2 into human cells. (**A**) Structure of SARS-CoV-2; (**B**) structure of the S protein. S1 includes an NTD and RBD. S2 plays a role in promoting cell entry in membrane fusion and contains an FP domain, two seven-step repeat domains (HR1 and HR2), a TM domain, and a CT domain; (**C**) schematic representation of the infection of SARS-CoV-2 into human cells and toxic effects. S protein: spike protein; NTD: N-terminal domain; RBD: receptor-binding domain; FP: fusion peptide; TM: transmembrane; CT: C-terminal; SARS-CoV-2: severe acute respiratory syndrome coronavirus 2.

The coronavirus (CoV) genome ranges in length from 26 to 32 kilobases. It is the largest known viral RNA genome. Until now, seven types of CoVs that can cause fatal respiratory tract infections have been identified [21]. Among these, two are classified as α-CoV (229E and NL63) and five as β-CoV (OC43, HKU1, SARS-CoV, SARS-CoV-2, and Middle East Respiratory Syndrome (MERS)-CoV) [22]. SARS-CoV-2 belongs to lineage B of the β-CoVs, a subgroup of Sarbecovirus. SARS-CoV-2 is an enveloped, large, and positive-sense single-stranded RNA virus. It shares an 80% sequence identity with SARS-CoV and a 50% sequence identity with MERS-CoV. The genome of SARS-CoV-2 comprises 14 open reading frames that encode 27 proteins. These include major structural proteins: the spike (S) protein, membrane (M) protein, envelope (E) protein, and nucleocapsid (N) protein (Figure 1A).

ACE2 is responsible for allowing viral entry into cells via interactions with the S protein [23]. The S protein includes the S1 and S2 subunits, with the receptor-binding domain (RBD) on the tip of the S1 subunit. The proprotein convertase (PPC) site is discovered through the S-protein gene sequence of SARS-CoV-2, and it is the cutting point between S1 and S2 of SARS-CoV-2 (Figure 1B) [24]. Receptor binding induces the dissociation of S1 and ACE2, which elongates the structure of the S2 subunit so that the fusion peptide on it attaches to the host cell from the sub-fusion state to the fusion state. This sequence of events drives human infection. Another cellular factor important for viral entry is transmembrane protease serine 2 (TMPRSS2), which can cleave the protease ADM117 of ACE2 and activate the S protein for membrane fusion [25]. ACE2 and TMPRSS2 are mainly expressed in epithelial cells. Of these lung cells, 6.7% of type II cells express ACE2 and 3.8% co-express ACE2 and TMPRSS2 [26].

SARS-CoV-2 uses ACE2 as its receptor and mainly infects ciliated bronchial epithelial cells and lung cells [27]. When SARS-CoV-2 enters the respiratory tract, it irritates and damages tracheal mucosal cells. The resulting symptoms include coughing, fevering, fatiguing, etc. Along with the severe infections, especially after the gradual damage to the bronchioles and alveoli, the surrounding connective tissues can also be damaged due to inflammation. This can result in the leak of a large amount of protein-rich inflammatory exudate into the alveoli, occupying the space that normally holds air. Consequently, a carbon dioxide and oxygen exchange is difficult in the limited space, directly leading to difficulty in breathing and serious and life-threatening damage to the liver, kidney, nervous system, and other organs (Figure 1C) [28,29,30,31,32,33,34,35].

In the initial stage, the condition of SARS-CoV-2 patients is not serious. Later, failure of multiple organs may occur, and the severity of the disease may be related to the cytokine storm [36]. The specific molecules involved are still unclear.

## 3. Potential Therapeutic Strategies

**Figure 2:**
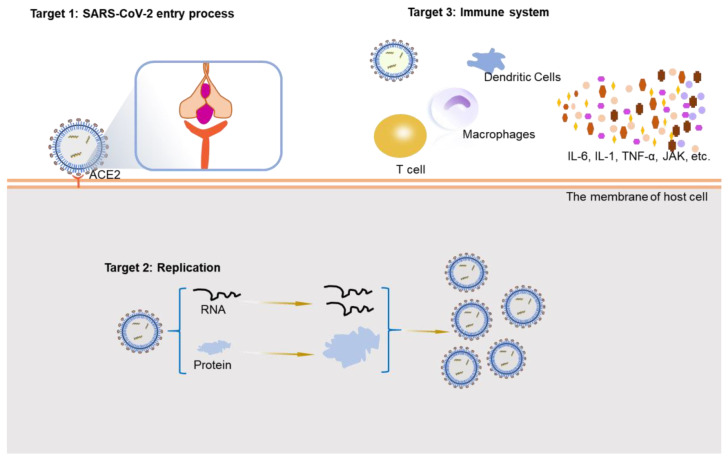
Potential therapeutic targets for the treatment of COVID-19. ACE2: angiotensin-converting enzyme 2; RNA: ribonucleic acid.

COVID-19 is a public health emergency of global concern. A large number of SARS-CoV-2-infected patients exhibit a mild-to-moderate illness, about 5–10% of the infections occur serious and life-threatening cases, and the mortality rate is about 2% [37]. The number of new confirmed patients continues to increase, highlighting the importance of the development of rapid and effective therapeutic strategies by the design of new specific drugs or the repurposing of existing drugs [38]. Herein, we summarize the therapeutic effects of the existing drugs and substances from three aspects based on potential targets: virus entry, virus replication, and the immune system (Figure 2). We also update the latest research on potential therapeutic strategies to provide new methods and ideas for future treatment methods development.

### 3.1. Entry of SARS-CoV-2

The S protein of SARS-CoV-2 binds to ACE2 in host cells to form the S protein–ACE2 complex. The complex is internalized by endocytosis and facilitates the entry of each virion into the cytoplasm. Inhibiting the binding of the S protein and ACE2 receptor to block the entry of SARS-CoV-2 into the host cells is an attractive approach.

#### 3.1.1. S Protein of SARS-CoV-2

**Figure 3:**
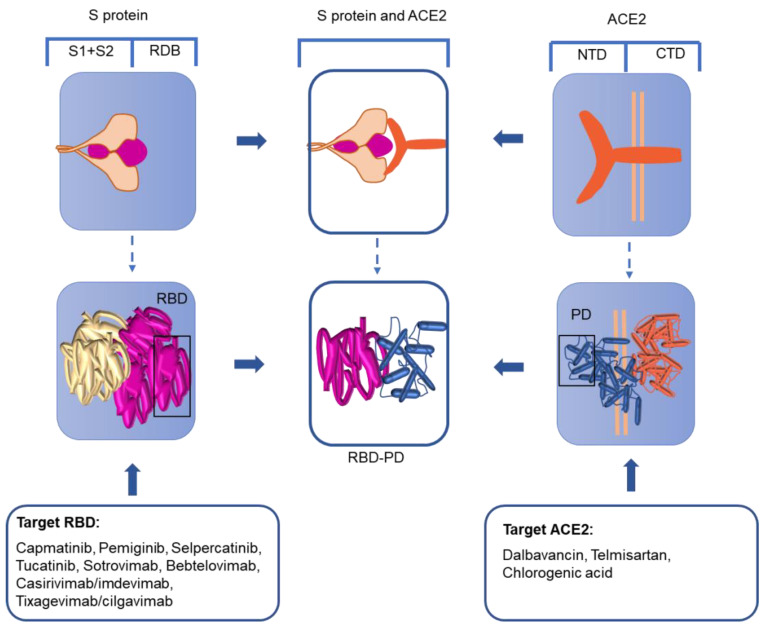
S protein and RBD process. S protein: spike protein; RBD: receptor-binding domain; ACE2: angiotensin-converting enzyme 2; PD: protease domain; NTD: N-terminal domain; CTD: C-terminal domain.

The trimer-shaped S protein with its’ S1 and S2 subunits on the surface of the virus harbors an RBD in the S-protein fusion active center [39]. When the S protein is fused, it undergoes structural reorganization with the RBD moving upward, leaving the area for receptor binding [40]. In this process, the S1 subunit falls off and the S2 subunit adopts the fused state [41,42,43]. Therefore, based on the idea that the S-protein RBD is the key binding site in human infection, a variety of strategies for the treatment of SARS-CoV-2 have been proposed (Figure 3).

Anticancer drugs may be beneficial for the treatment of SARS-CoV-2. Of the Food and Drug Administration (FDA)-approved anticancer drugs, four drugs (capmatinib, pemiginib, selpercatinib, and tucatinib) completely dock with the S protein of SARS-CoV-2. The docking prevents receptor binding, preventing virus infection [44].

Monoclonal antibodies (mAbs) therapy is another efficient approach for the treatment of SARS-CoV-2, given the 17 residue positions for antibodies that have been discovered in the RDB [45]. Various monoclonal antibodies have been screened for the RDB target of the S protein [46,47,48]. Sotrovimab is a recombinant human monoclonal immunoglobulin GI antibody that targets RBD epitopes outside the receptor-binding motif [49,50]. A COMET-ICE clinical trial demonstrated an 85% reduction in the primary endpoint of hospitalizations (more than 24 h) or death for those receiving sotrovimab compared to placebo. On 26 May 2021, sotrovimab was approved by the FDA for the treatment of mild and moderate symptoms of CoV-2 in people 12 years and older [51]. However, the efficacy of sotrovimab for the different new mutant strains would remain to be studied. Recently, the Omicron variant has become the dominant variant of SARS-CoV-2 globally, and bebtelovimab, which was shown to be effective against the different Omicron subvariants, was authorized in adults, those aged 12 years or older, and pediatric patients [52,53]. The emergence of virus-escape mutants and the development of antibody resistance have made the combined use of two or more antiviral monoclonal antibodies against different epitopes a critical aspect to improve efficacy. Casirivimab/imdevimab (REGN-COV2, Ronapreve), a cocktail of human antibodies against the S-protein RBD of SARS-CoV-2, was first authorized by the FAD for emergency use to treat patients with mild-to-moderate COVID-19 [54,55]. Evusheld (tixagevimab/cilgavimab), isolated from COVID-19 patients, can bind to different locations of the receptor-binding region of the SARS-CoV-2 S protein to neutralize SARS-CoV-2 [56,57]. These mAbs have indicated high effectiveness in trial studies with a decrease of 70–85% in hospitalization or death [58].

Nanobodies, which are composed of single-heavy-chain dimers, were first discovered in the serum of camels [59]. They are smaller, more soluble, stable, and permeable, with a higher affinity compared to traditional antibodies [59,60]. Nanobodies in the alpaca heavy-chain antibody library can bind to the RBD of the S protein, as demonstrated by surface plasmon resonance. The binding site is the same as that of ACE2. A stable nanobody–RDB complex is generated via a hydrogen bond or bivalent salt linkage with RBD residues. The complex can neutralize SARS-CoV-2 and has preventive and therapeutic effects on virus infection [61,62]. Wu et al. prepared a SARS-CoV-2 nanobody that can simultaneously bind to the RBD to block human infection [63].

An aptamer is a single-stranded oligonucleotide. The oligonucleotide is small, safe, and has been widely used to treat COVID-19. The ideal aptamer has excellent structural dynamics and blocking behavior, ensuring optimal affinity to the RBD of SARS-CoV-2. The prevention and treatment prowess of the aptamer rely on its ability to compete with ACE2. Aptamer CoV2-6 can compete and substitute with ACE2 in binding to the S-protein RBD. To improve the stability, affinity, and inhibition efficacy, CoV2-6 was further shortened and engineered as a circular bivalent aptamer, termed CoV2-6C3 [64]. Other oligonucleotide aptamers containing a conserved sequence motif that can block the interaction of the RBD-ACE2 have been designed to prevent infection [65].

#### 3.1.2. ACE2 Receptor for SARS-CoV-2

ACE2 is comprised of an extracellular-facing N-terminal domain and a C-terminal transmembrane domain with a cytosolic tail. The N-terminal portion of the protein contains the claw-like protease domain (PD), which can recognize the SARS-CoV-2 RBD via polar residues. The C-terminal domain is termed the Collectrin-like domain. The SARS-CoV-2 RBD combines with the ACE2 PD to form an RBD-PD complex (Figure 3). Therefore, as the receptor of SARS-CoV-2, ACE2 is a possible therapeutic target that needs to be studied in depth.

In the treatment of SARS-CoV-2, in addition to drugs and antibodies developed against the virus target, drugs with a high affinity to ACE2 have also been discovered. Dalbavancin, in the peptide drug library, can directly bind with ACE2 through the action of ACE2 amino acids. This binding prevents ACE2 from interacting with the viral S protein [66]. Although dalbavancin has not been extensively investigated in clinical trials, there is great hope for its future application. Telmisartan has been evaluated in clinical trials as an angiotensin receptor blocker [67]. Chlorogenic acid can stably bind to the Glu329/Gln325 and Gln42/Asp38 sites in ACE2 as a potential inhibitor of COVID-19 [68]. Researchers envision administering an antibody or single-chain antibody fragment that can bind the host cell membrane ACE2 protein and inhibit the interaction of the S protein with ACE2 [69]. OM-85 (a standardized lysate of human airway bacteria) and B2R (the kinin B2 receptor) are antagonists that can reduce the expression level of ACE2 in humans and prevent the transmission of signaling pathways [70]. Wei et al. reported that the exogenous supplementation of ACE2 via mesenchymal stem cells (MSCs) had better therapeutic effects, which might be due to the increased activities of the secretory ACE2 [71]. Despite the increasing attention paid to ACE2, there is scant literature on the therapeutic strategies and efficient drugs that target ACE2. Further information is critical to establish effective prevention and control actions.

### 3.2. Preventing SARS-CoV-2 Reproduction

**Figure 4:**
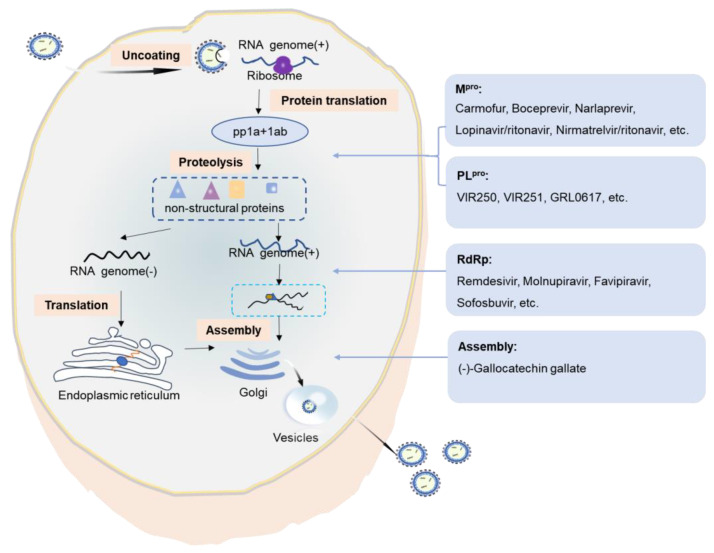
Replication of SARS-CoV-2 in host cells. M^pro^: main protease; pp1a: polyprotein 1a; pp1ab: polyprotein 1ab; PL^pro^: papain-like proteinase; RNA: ribonucleic acid; RdRp: RNA-dependent RNA polymerase.

SARS-CoV-2 enters the host cell and replicates in the target cells. The SARS-CoV-2 genome is large and harbors two open reading frames (1a and 1b) at the 5′ end. These encode two large polyproteins, polyprotein 1a (pp1a) and polyprotein 1ab (pp1ab). The two polyproteins are cleaved and translated into a mature non-structural protein (NSP), which is responsible for the replication and transcription of the sub-genomic RNAs. The main protease, the 3C-like serine-type protease (3CL^pro^ or M^pro^) encoded by NSP5 and the papain-like proteinase (PL^pro^) encoded by NSP3, are important in the cleavage of polyproteins. PL^pro^ cleaves the NSP1/2, NSP2/3, and NSP3/4 boundaries. The RNA-dependent RNA polymerase (RdRp; also known as NSP12) is a core component of the virus replicase–transcriptase complex responsible for the replication and transcription of viral RNA. M^pro^, PL^pro^, and RdRp have indispensable roles in SARS-CoV-2 replication and are promising targets for drug design (Figure 4).

M^pro^ is essential for the cleavage of the viral polyprotein pp1ab at 11 discrete sites [72]. The released NSPs form a replicase–transcriptase complex, which in turn is responsible for viral replication [73]. The antineoplastic drug carmofur and anti-hepatitis C virus (HCV) drug boceprevir are approved drugs that are capable of inhibiting SARS-CoV-2 by targeting M^pro^ [74]. The carbonyl-reactive group of carmofur and trichostatin A can covalently bind to catalytic Cys145, while boceprevir occupies the substrate-binding pocket of M^pro^ and combines with the catalytic Cys145 to form a covalent bond [75,76]. Narlaprevir is a potent second-generation drug based on boceprevir. It inhibits the HCV NS3 protease and also has moderate inhibitory activity against SARS-CoV-2 M^pro^ by binding to the active site of M^pro^ through a C-S covalent bond with catalytic Cys145 [74]. 5-((1H-Imidazol-1-yl) methyl) quinolin-8-ol (DD1) is a derivative formed by the action of chloroquine and hydroxychloroquine. DD1 binds to the active residues in M^pro^ 6Y84 (GLN127, PHE223, and others) through hydrogen bond affinity. This can hinder the activity of the M^pro^ and inhibit the translation process [77,78]. Three modified dipeptides (11a, 11b, and 13b) were designed to inhibit the activity of M^pro^. These compounds covalently block Cys145 by the formation of hemithioacetal/ketal [78,79]. The bioavailability of 11a was 87.8% in animal experiments [78]. Recently, two novel bicycloproline-containing M^pro^ inhibitors (MI-09 and MI-30) with excellent antiviral activity in cell-based assays were designed and synthesized. The inhibition modes of MI-09 and MI-30 are similar to that of 11a [80]. The only difference is that these compounds contain two cycloproline moieties, which obviously increase the exposure of the drugs in the body. Besides covalent inhibitors, baicalein is identified as a non-covalent inhibitor of SARS-CoV-2 M^pro^. Baicalein occupies the core of the substrate-binding pocket, acting as a ‘shield’ in front of the catalytic dyad Cys145 to prevent the peptide substrate approaching the active site [81,82]. Through a combination of a structure-based drug design, Jin et al. analyzed over 10,000 compounds and 6 of those compounds (ebselen, shikonin, tideglusib, PX-12, disulfiram, and carmofur) were shown to inhibit M^pro^ [83]. Moreover, some drugs developed to treat HIV infections were studied as potential agents for COVID-19 [84]. Lopinavir/ritonavir (LPV/r), which is a prescription medicine approved by the FDA for the treatment of HIV infection, was proposed as a candidate agent due to its well binding to the SARS-CoV-2 M^pro^ [85]. In addition, nirmatrelvir/ritonavir (Paxlovid^®^) was approved on December 24, 2021, by the FDA for the emergency treatment of COVID-19 [86,87,88]. Nirmatrelvir inhibits the protein cleavage reaction of the M^pro^ of SARS-CoV-2, thereby inhibiting viral replication. Ritonavir acts as a booster by inhibiting a drug-metabolizing enzyme cytochrome p450 [89]. Paxlovid^®^ would reduce the risk of hospitalization or death in 90% of subjects with mild-to-moderate disease [90].

SARS-CoV-2 PL^pro^ is responsible for the cleavage of the N-termini of replicase polyproteins, which is critical in protein maturation. Compared with SARS-CoV-2 M^pro^, there are fewer studies on SARS-CoV-2 PL^pro^ inhibitors. Rut et al. used a combinatorial substrate library to perform a comprehensive activity profiling of SARS-CoV-2 PL^pro^. Two inhibitors (VIR250 and VIR251) designed to target SARS-CoV-2 occupy the S4-S1 pockets of the substrate-binding site, with high selectivity for SARS PL^pro^ [91]. Naphthalene-based derivatives also inhibit SARS-CoV-2 replication by binding to the residues in the BL2 ring of PL^pro^ [92]. A screen of a library of approved compounds revealed the activity of GRL0617 in preventing SARS-CoV-2 infection by binding to the catalytic domain and inhibiting the deacylation activity of PL^pro^ [93]. Multiplexed enhanced protein dynamics (MePROD) revealed that the translation inhibitors cycloheximide and emetine can significantly inhibit the replication of SARS-CoV-2 [94]. MePROD can determine the changes in the proteome and translator in human cells. Cycloheximide is a translation extension inhibitor that can bind to the E site to block eEF2-mediated tRNA translocation. Emetine is a 40S ribosomal protein S14 inhibitor that inhibits the formation of peptide chains [95].

In addition, several drugs targeting virus RdRp have shown therapeutic potential against SARS-CoV-2. These drugs include favipiravir [96], sofosbuvir [97], ribavirin [98], galidesivir [99], remdesivir [100], molnupiravir [101], and so on. Among those, remdesivir, which was initially used to treat Ebola virus infections [100,102], is the first approval of the FDA for the emergency treatment of COVID-19 [103]. Remdesivir leads to the retention of the RNA 3ʹ-nucleotide at the substrate-binding site of the RNA polymerase, which interferes with the entry of the next nucleoside triphosphate and consequently inhibits RNA polymerization [104,105]. Up to now, although the drug has been approved for severe patients, the benefits of remdesivir for the treatment of COVID-19 remain debated as some clinical trials have shown discordant results [106,107,108]. The WHO recommends against the use of remdesivir for all patients, based on the results of the solidarity trial, which failed to observe the improvement in clinical mortality [109,110]. A meta-analysis concluded that there is a high probability that remdesivir reduces mortality for nonventilated patients with COVID-19 requiring supplemental oxygen therapy [107]. Similarly, molnupiravir, a prodrug for the ribonucleoside analogue β-d-N4-hydroxycytidine (NHC; EIDD-1931), has recently been approved in some countries for the patients with mild-to-moderate COVID-19 [18,111]. It was the first approval for oral drugs which have a major advantage over injectable drugs (remdesivir) against COVID-19 [112]. Suramin has stronger electronegativity than RNA polymerase and so can compete for binding to the gene sequence of the virus [113]. Suramin occupies two sites for RNA replication. One site prevents the primer from binding to the template strand [114]. The other site prevents the extension of the primer chain and hinders ATP from entering the catalytic site to provide energy [115]. Suramin is 20 times more powerful than remdesivir [114]. However, it is difficult to determine if the clinical benefits of suramin outweigh its toxic effects [116]. The suramin derivative NCTU-Alan-2026 is a small molecule that has less toxic effects. It is designed and synthesized to block protein interactions to inhibit cell proliferation [117]. The development of NCTU-Alan-2026 has provided new insights for the future development of antiviral drugs. Clofazimine also inhibits RNA replication by affecting the unwinding of RNA polymerase (NSP13) [118], which is essential for the CoV replication [119]. Interestingly, as an inhibitor of SARS-CoV-2 RdRp, Zn(II) may be a promising candidate, with direct inhibitory effects on the replicative cycle of SARS-CoV-2 [120]. In addition, some molecular docking studies showed that tenofovir and lamivudine, which are widely used to treat HIV infection, could be used effectively against SARS-CoV-2 [84].

In particular, during the replication process of SARS-CoV-2 in host cells, membrane and membrane-less organelles are formed to ensure the smooth progress of various biochemical processes [121]. Liquid–liquid phase separation (LLPS) is the basis for the formation of membrane-less organelles [122]. Protein and nucleic acid are condensed into a liquid-like condensate through LLPS and are assembled or disassembled in a spatially and temporally controlled manner [123]. Based on these findings, (−)-gallocatechin gallate, a green tea polyphenol, was recently discovered to efficiently prevent SARS-CoV-2 replication by blocking the LLPS of the N protein caused by RNA and affecting RNA and protein assembly [124].

### 3.3. Immunomodulatory Effects

**Figure 5:**
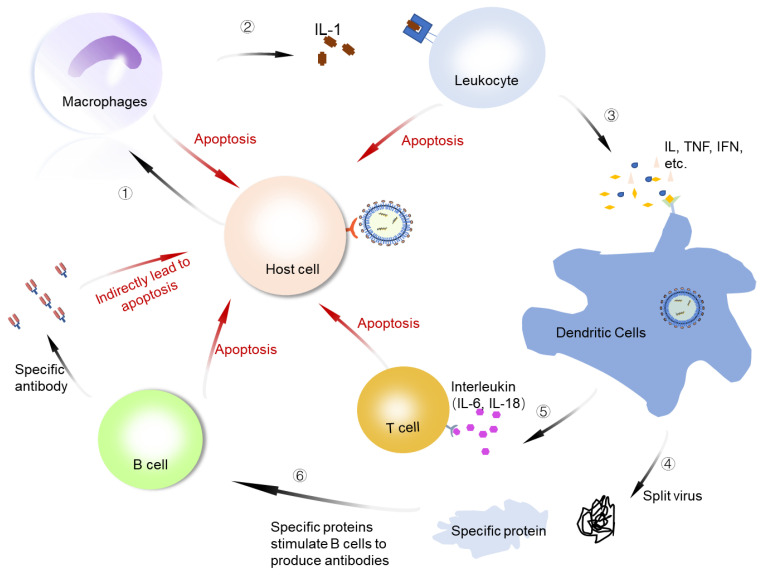
Schematic representation of cytokine storm. ① A large number of viruses infect host cells and stimulate macrophages to fight the virus. ② Phagocytes can secrete cytokines to attract white blood cells to inhibit SARS-CoV-2. ③ Leukocytes secrete specific cytokines to stimulate cell pathways to attract the arrival of dendritic cells to reduce the number of viruses. ④ Dendritic cells degrade the virus into specific proteins and nucleic acids. ⑤ Dendritic cells also secrete IL-12 and IL-18, which activate signal molecules to stimulate T-cell proliferation and enhance immune effects. ⑥ Virus-specific proteins and nucleic acids induce B cells to differentiate into effector B cells that can secrete specific antibodies to fight the virus. IL: interleukin; IFN: interferon; TNF: tumor necrosis factor.

An important feature of COVID-19 is immune regulation disorder, which manifests as excessive inflammation. When SARS-CoV-2 infects the human body, it stimulates the body’s immune response line of defense. There are no serious clinical manifestations in the early stage of COVID-19 in patients who become critically ill or die. In the late stage, the viruses in the human body stimulate immune cells that include macrophages, dendritic cells, T cells, and white blood cells to activate anti-inflammatory factors that jointly respond to the invasion of viruses (Figure 5). The proinflammatory feed-back loop caused by SARS-CoV-2 infection could generate a massive production of chemokines, which orchestrate immune cell infiltration and the secretion of cytokines. The rapid rise of the concentrations of cytokines within a short period of time leads to a cytokine storm and acute respiratory distress syndrome. Some possible immune signatures in patients with COVID-19 and the targets for the treatment of the disease have been proposed. These include janus kinase (JAK), interleukins, tumor necrosis factor (TNF), and others [125]. The cytokine storm disrupts the immune function and is an important cause of severe illness and death. The production of SARS-CoV-2 symptoms is not clear, but it is closely related to specific cytokines [126,127]. The effective suppression of the cytokine storm should be an efficient way to prevent severe disease and death.

Clinical observations indicate that COVID-19 patients manifest an acute elevation of serum levels of inflammatory mediators, such as IL-6, IL-1, TNF-α, and JAK [128]. These elevations are associated with disease severity and progression. Therefore, the therapeutic strategies targeting IL-6, IL-1, TNF-α, and JAK have been proposed to quell the cytokine storm caused by SARS-CoV-2. A series of biologics have been designed that target IL-6, a key cytokine in the mediation of fever and the acute-phase response (Table 1). Among those, tocilizumab, which has been mainly used for the treatment of rheumatoid arthritis [129], is suggested to have beneficial effects in reducing the inflammatory response in severe COVID-19 patients. A meta-analysis of randomized clinical trials showed that tocilizumab was associated with a lower mortality rate in COVID-19 patients [130]. Moreover, the intravenous and subcutaneous forms of administration of tocilizumab almost have the same stability in treating patients with COVID-19 [131,132,133]. Sarilumab, a high-affinity anti-IL-6 receptor antibody, which has been approved for rheumatoid arthritis treatment by the FDA, has also showed efficiency in patients with COVID-19 [134]. Gremese et al. studied 53 patients who were treated with sarilumab, and the results showed that 89.7% of the patients significantly improved, 70.6% were discharged from the hospital, and 85.7% no longer needed oxygen therapy [135]. In addition, the same results were concluded by Benucci et al. [136]. Sarilumab is considered as an alternative regimen for the treatment of COVID-19 [137]. However, recently, some randomized trials showed that sarilumab treatment did not improve outcomes in patients with moderate-to-severe COVID-19 pneumonia [138,139]. So, further clinical studies with larger sample sizes and long-term follow-up are needed to assess the efficacy and safety of this drug. In addition, some traditional Chinese medicine, such as *Glycyrrhizae Radix et Rhizoma (Gancao)* and *Pinelliae Rhizoma (Banxia)*, may act by suppressing the IL-6 amplifier and have been used to treat some moderately ill COVID-19 patients [140]. IL-1 is another important cytokine associated with inflammation. It is mainly produced by the activated mononuclear phagocytes. IL-1 can induce other proinflammatory cytokines, such as IL-6 and TNF-α. Several inhibitors that target IL-1, including canakinumab and anakinra, are effective for the treatment of the cytokine storm (Table 1) [141]. One report described patients from Saint Joseph’s hospital in Paris, France, who received anakinra treatment. The mortality rate and the need for invasive mechanical ventilation in the ICU were clearly reduced [142].

TNF-α is a key inflammatory factor that triggers a cytokine storm and is involved in mediating the inflammatory response in acute respiratory distress syndrome. Therefore, TNF-α inhibitors are expected to have promising therapeutic effects. A variety of TNF-α inhibitors, including adalimumab, etanercept, vitamin D, emapalumab, and infliximab, have been designed. Their effectiveness has not been proven in actual treatment (Table 1). JAK is an intracellular tyrosine kinase that mediates signals from cytokines, hormones, and growth factors. By blocking cytokine signaling and reducing excessive inflammatory responses, JAK inhibitors play an important role in the control of the cytokine storm in COVID-19 patients. Several JAK inhibitors, including baricitinib, ruxolitinib, tofacitinib, and fedratinib, are being studied for the treatment of severe COVID-19 (Table 1). Recently, two large clinical trials of baricitinib, involving 2558 patients hospitalized with COVID-19, showed significant clinical and survival benefits. Baricitinb has been recommended for clinical practice by multiple guidelines [143].

In addition to IL-6, IL-1, TNF-α, JAK, and other inflammatory mediators, such as the granulocyte-macrophage colony-stimulating factor (GM-CSF) and the complement protein C5, have been developed as therapeutic targets against the cytokine storm caused by SARS-CoV-2. GM-CSF is a cytokine with a cardinal role in the modulation of inflammation. Mavrilimumab is an anti-GM-CSF receptor-α monoclonal antibody that has improved clinical outcomes in patients with COVID-19. Earlier discharge from the hospital and no progression to death after mavrilimumab treatment were described [144]. Moreover, the complement system may be a valuable target for COVID-19 therapy, as it is an integral component of the innate immune response to virus infection. Eculizumab, a humanized mAb with a high affinity to complement factor C5, is developed for use in the treatment of COVID-19 [145,146].

**Table 1:** Immunosuppressive agents are used to treat COVID-19 inflammatory phase.

Target and Drugs	Initial Target	Administration Mode	Results	References
**IL-6**				
Tocilizumab	Rheumatoid arthritis	Injection	Reduced mortality.	[147]
*Babaodan*	Infectious viral hepatitis	Oral	A large number of IL-6 and C-reactive proteins were eliminated in patients.	[148]
Methylprednisolone	Rheumatic disease	Injection	Patients with oxygen saturation improved rapidly and survival time became longer.	[149]
Olokizumab	Rheumatoid arthritis	Injection	The body temperature dropped to normal levels.	[150,151]
Sarilumab	Rheumatoid arthritis	Injection	A total of 89.7% of inpatients experienced significant improvement in symptoms and 70.6% were discharged.	[135]
Siltuximab	Castleman’s disease	Injection	Reduced mortality.	[152]
Clazakizumab	Rheumatoid arthritis	Injection	Symptoms, oxygen demand, radiologic findings, and inflammatory indicators improved markedly.	[153]
**IL-1**				
Canakinumab	Rheumatologic disorders	Injection	Improved oxygenation, decreased serum C-reactive protein levels.	[154]
Anakinra	Rheumatoid arthritis	Injection	Relieved clinical signs in critically ill patients.	[155,156]
**TNF-α**				
Infiximab	Rheumatoid arthritis	Injection	Reduced serological response to SARS-CoV-2.	[157,158]
Etanercept	Psoriatic arthritis	Oral	Delayed the development of olfactory and taste dysfunction in patients.	[159,160]
Adalimumab	Rheumatoid arthritis	Injection	Radiological improvement of the lung without any complications.	[161,162]
Vitamin D	Anti-rickets	-	Inhibited the expression of proinflammatory cytokines and reduced viral load.	[163]
Emapalumab	Hemophagocytic	-	Effective in a refractory, persistent, and progressive cytokine storm.	[164]
**JAK**				
Ruxolitinib	Myelofibrosis	Oral	The cytokine content was reduced, and the patient’s chest CT improved by 80%.	[165]
Fedratinib	Myeloproliferative tumor	-	Reduced patient mortality.	[166]
Tofacitinib	Rheumatoid arthritis	Injection	Respiratory symptoms were relieved after 5 days.	[167]
Baricitinib	Rheumatoid arthritis	Oral	Accelerate improvement in clinical status and reduce 28-day mortality.	[168,169,170]
**IL (IL-1, IL-3, IL-2, IL-5, IL-6, IL-8) and TNF-α**				
Glucocorticoid	SARS; MERS	Injection	Significantly reduced risk of death by 62%.	[171,172]
Lidocaine/dexamethasone	-	-	Showed anti-inflammatory capacity on SARS-CoV-2-triggered immune pathways.	[173]
Fluvoxamine	Antidepressant	Injection	Reduced the need for advanced disease care in this high-risk population.	[174]
**IL-6, IL-8, TNF-α**				
Azithromycin	Macrolide antibiotics	Oral	Low mortality.	[175,176]
Melatonin	Immune system	-	Effectively improved inflammation.	[177]
Cyclosporine A	Hepatitis B virus	-	Reduced mortality in patients with moderate-to-severe disease.	[178]
**GM-CSF**				
Mavrilimumab	Refractory rheumatoid arthritis	Injection	Improved clinical outcomes, well tolerated without discomfort.	[144]
**C5**				
Eculizumab	Orphan disease	Injection	Reduced inflammatory responses and improvedlung function and lymphocyte recovery.	[145,146]

Abbreviations: TNF-α, tumor necrosis factor-α; IL, interleukin; JAK, Janus kinase; GM-CSF, granulocyte–macrophage colony-stimulating factor; CCR5, C–C chemokine receptor type 5.

## 4. Conclusions and Perspectives

The development of effective treatments for SARS-CoV-2 remains an urgent priority. This review summarizes possible treatment strategies that target the entry of the virus, virus replication, and the immune system. The aim is to inform the development of efficacious prevention and treatment strategies. The consideration of targeted treatment to prevent SARS-CoV-2 entry into human cells and the development of symptoms will hopefully bring novel insight into the treatment of COVID-19.

Some antiviral drugs have specific effects against SARS-CoV-2. The efficacy and safety of these promising candidate drugs in the treatment of SARS-CoV-2 need to be confirmed. Moreover, some monoclonal antibodies have demonstrated efficacy against COVID-19 and have been approved in some countries for the treatment and prevention of COVID-19, but the emergence of variant lineages is now one of the most difficult obstacles to controlling the COVID-19 pandemic.

The infection by SARS-CoV-2 may cause disseminated intravascular coagulation and venous thromboembolism in patients with some critically ill disease. Some research has demonstrated that anticoagulant therapy, mainly with low-molecular-weight heparin, has been associated with better outcomes in severe COVID-19, especially in the early treatment [179,180]. However, the use of anticoagulation or high doses of heparin is still controversial.

In addition, several studies showed the use of immune-suppressive drugs combined with the natural immune suppression caused by the virus can lead to increasing secondary bacterial and fungal infections [181,182,183], but more research about antimicrobial resistance and its correlation with antibiotic misuse in COVID-19 patients is required [184]. Moreover, the high rate of multidrug-resistant organisms among hospitalized patients alerted that it is urgent to use the antibiotics with a higher potential for resistance and to evaluate trends in antibiotic resistance [185].

With the rapid spread of SARS-CoV-2, as well as the rising global mortality rate of COVID-19, research has focused on the development of potential vaccine candidates for protection against SARS-CoV-2. To date, the total number of vaccine doses administered globally has reached 90.9 billion. Along with the virus spread, several mutant strains have emerged. These mutant strains may result in epitope changes that reduce the affinity of antibodies produced by neutralizing antibody therapy or vaccines. Therefore, developing effective, specific, and general antiviral drugs is crucial to end the COVID-19 pandemic and to prevent future social and economic hardships.

## Data Availability

Not applicable.
